# Variations of rhizospheric soil microbial communities in response to continuous *Andrographis paniculata* cropping practices

**DOI:** 10.1186/s40529-020-00295-1

**Published:** 2020-06-15

**Authors:** Junren Li, Xiuzhen Chen, Simin Li, Zimei Zuo, Ruoting Zhan, Rui He

**Affiliations:** 1 Research Center of Chinese Herbal Resource Science and Engineering, Guangzhou University of Chinese Medicine, Guangzhou, 510006 People’s Republic of China; 2 Key Laboratory of Chinese Medicinal Resource from Lingnan (Guangzhou University of Chinese Medicine), Ministry of Education, Guangzhou, 510006 People’s Republic of China

**Keywords:** *Andrographis paniculata*, Continuous cropping, Rhizospheric soil, Microbial community, High-throughput sequencing

## Abstract

**Background:**

Changes of soil microbial communities are one of the main factors of continuous cropping problem. *Andrographis paniculata* has been reported to have replant problem in cultivation. However, little is known about the variations of rhizosphere soil microbial communities of *A. paniculata* under a continuous cropping system. Here, Illumina MiSeq was used to investigate the shifts of rhizospheric bacterial and fungal communities after continuous cropping of *A. paniculata*.

**Results:**

The bacterial diversity increased whereas the fungal diversity decreased in rhizosphere soil after consecutive *A. paniculata* monoculture; and the soil microbial community structure differed between newly plant soil and continuous cropped soil. Taxonomic analyses further revealed that the bacterial phyla *Proteobacteria*, *Acidobacteria* and *Bacteroidetes* and the fungal phyla *Zygomycota*, *Ascomycota* and *Cercozoa* were the dominant phyla across all soil samples. The relative abundance of phyla *Acidobacteria* and *Zygomycota* were significantly increased after continuous cropping. Additionally, the most abundant bacterial genus *Pseudolabrys* significantly decreased, while the predominant fungal genus *Mortierella* increased considerably in abundance after continuous cropping.

**Conclusions:**

Our results revealed the changes on diversity and composition of bacterial and fungal communities in rhizospheric soil under continuous cropping of *A. paniculata*. These data contributed to the understanding of soil micro-ecological environments in the rhizosphere of *A. paniculata*.

## Background

Continuous cropping is the practice of cultivating the same crop in soil that had previously supported the same plant year after year without rotation with other crops (Shipton 1977), which sometimes leads to yield reduction, quality deterioration, poor growth status and disease aggravation (Zhou et al. 2011; Liu et al. 2014). It is frequently observed in medicinal plants such as *Panax ginseng* (Ying et al. 2012), *Panax notoginseng* (Dong et al. 2016), *Rehmannia glutinosa* (Wu et al. 2018a), etc. The complex mechanisms of replant disease have been considered including many factors such as imbalance of soil nutrients, built up of soil-borne pathogens, autotoxicity from root exudates, and the shifts in microbial community (Fuentes et al. 2009; Huang et al. 2013). Recently, increasing evidences have speculated that changes of the soil microbial community may contribute to replant problem in some plants, and the diversity and composition of soil microbial communities also significantly affected by continuous cropping systems (Ying et al. 2012; Dong et al. 2016; Wu et al. 2018a, 2018b; Li et al. 2019a). As well known that, soil microorganisms play crucial roles in the cycling of plant nutrients, the energy flow of either natural or anthropogenically altered soils, and the maintenance of soil ecosystem (Waldrop et al. 2000; Nannipieri et al. 2017). High microbial diversity and appropriate composition contribute to maintaining soil health and promoting plant growth. Additionally, there are interactions between plant roots and soil microorganisms. Soil bacteria and fungi both directly and indirectly affect the health of plants, and plants roots exert strong influences on rhizospheric soil microorganisms through producing exudates as well as secondary metabolites (Haldar and Sengupta 2015). These interactions participate in plants’ fitting the natural environments including the continuous cropping system. Thus, revealing the diversity and composition of microbial community in continuously cropped soil may help better understanding continuous cropping in medicinal plants.

*Andrographis paniculata* (Burm. f.) Nees is an important traditional herb in Southern and Southeastern Asian countries (Raina et al. 2013). It exhibits various pharmacological activities and is commonly used for its antipyretic, antibacterial, antimalarial, anti-inflammatory, anti-thrombogenic, hepatoprotective effects in several traditional medicine systems worldwide (Abu-Ghefreh et al. 2009; Saxena et al. 2010; Bardi et al. 2014). Documented in Chinese Pharmacopoeia, *Andrographis herba* (*Chuanxinlian*), the dried aerial parts of *A. paniculata* is used to remove pathogenic heat from the blood and toxic material from the body, reduce swelling in Chinese medicine system. Owing to its distinct curative effects, there is a vastly increasing demand of *A. paniculata*. In the commercially standardized cultivation, replant disease often occurs on *A. paniculata* and growers usually avoid the problem via crop rotation or leaving land unused; both approaches affect the production and quality of *Andrographis herba*. Our previous study also found that continuous cropping mainly lowered the quality of *Andrographis* herba, manifesting as decreased contents of main pharmacological active components (Li et al. 2019b). Some studies mainly emphasized on the allelopathy for the richness of allelopathic chemicals like terpenoids and flavonoids in *A. paniculata* (Li et al. 2010; 2014). However, little is known about the changes in bacterial and fungal communities that are associated with the continuous cropping of *A. paniculata* until now.

In this study, Illumina MiSeq sequencing was applied to investigate the changes of microbial communities in rhizospheric soil with 0, 1, and 2 years of *A. paniculata* consecutive monoculture histories. Our objective was to reveal changes in the diversity and composition of rhizospheric soil bacterial and fungal communities under a continuous cropping system of *A. paniculata*. The obtained results should be useful for understanding the effects of continuous cropping system on soil micro-ecology, and provide theoretical reference for better cultivation and management of *A. paniculata*.

## Methods

### Study sites and sampling

The experiments of *A. paniculata* continuous monoculture were carried out in a plastic sunshelter on “Shizhen Mountain” in Guangzhou University of Chinese Medicine, Guangzhou City, Guangdong Province, China (23^°^16′ N, 113^°^23′ E). This region has a typical subtropical monsoon climate with an average annual precipitation of 1800 mm and an average temperature of 21.9 ℃.

In the continuous cropping experiment, 150 seedlings were randomly allocated into three groups, AP0, AP1 and AP2 (one plant per pot, pot size was 25 cm height and internal diameter). In AP0 group, seedlings were planted with commercial horticultural soil with no prior planting of *A. paniculata* (the first-year planting in this study). Seedlings in the AP1 or AP2 group were planted with the same kind of soil that had 1-year, or 2-year *A. paniculata* cropping history (the second-year, or the third-year planting in this study), respectively. The commercial horticulture soil (Greenorchids horticultural Co. Ltd., Taiwan) was composted with peat soil, coir, perlite, vermiculite and carbonized rice husk; which contains 52% organic matter, 0.5% total nitrogen, 1% total phosphorus (indicates as  % of phosphoric anhydride in the soil) and 0.4% total potassium (indicates as % of potassium oxide in the soil). The continuous cropping experiment was performed during months of July–September under natural temperature and light, watering was conducted when plants needed, mainly according to the weather condition and same cultivation conditions were applied (Li et al. 2019b). After harvesting, the soil samples were collected from the three time-series groups respectively. For soil sampling, 30 pots with *A. paniculata* plants were randomly selected in each group; for each plant (in one pot), the aboveground part of the plant was cut and the soil surface coverings were removed. Then an iron cylinder (20 cm in depth and 2.5 cm in diameter) was inserted vertically from the top of soil taking the residual stem as the circular center, and the depth is 20 cm (regarded as one core). The roots inside the cylinder were taken out and then shaken to remove loosely attached soil, and the soil that remained tightly attached to the roots were brushed off and sampled as the rhizospheric soil. The rhizospheric soil collected from 15 cores were mixed and recorded as one sample, and each group contained two replicated samples; all soil samples were separately sieved (2 mm). In total, 6 soil samples were collected and stored at − 80 ℃ for DNA extraction.

### Soil DNA extraction, PCR amplification and high-throughput sequencing

Total soil DNA was extracted from 0.25 g soil using the Power Soil DNA Isolation kit (MOBIO Laboratories, Inc., USA) following the manufacturer’s instructions. After concentration and quality checking using a Nanodrop 2000 (Thermo Scientifics, USA), DNA samples were stored at −20 ℃ for PCR amplification. Each composite soil sample was extracted in triplicate, and the three successive DNA extractions of each sample were pooled before PCR to serve as a template. Primer sets of F338/R806 and ITS1F/ITS2 were used to amplify the V3-V4 region of the bacterial 16S rRNA gene and the fungal ITS region respectively as described (Crowther et al. 2014; Derakhshani et al. 2016). All PCR reactions were carried out with Phusion^®^ High-Fidelity PCR Master Mix (New England Biolabs, Ipswich, MA, USA) following the manufacturer’s instructions. The same volume of 1× loading buffer (containing SYBR green) was mixed with PCR products, and the samples were analyzed using 2% agarose gels for detection. Samples with bright main bands between 400 and 450 bp were selected for further analysis. The PCR products were mixed at equal density ratios, and then purified with Qiagen Gel Extraction Kit (Qiagen, Hilden, Germany). High-throughput sequencing was performed using Illumina MiSeq platforms at BIOMARKER Bioinformatics Technology Co., Ltd. (Beijing, China).

### High-throughput sequencing data analysis

After removing the adaptors and primer sequences, QIIME was used to assemble the raw sequences for each sample according to the unique barcode (Caporaso et al. 2010). Split sequences for each sample were merged using FLASH V1.2.7 (Magoc and Salzberg 2011). The sequences retained for each sample were processed following the established UPARSE pipeline (Edgar 2013). After removal of chimeras using the UCHIME method (Edgar et al. 2011), the high-quality sequences were assigned to operational taxonomic units (OTUs) using Mothur v.1.34.0 with a cutoff of 97% similarity and the most abundant sequence in each OTU was selected as the representative sequence. For species annotation, the bacterial representative sequences were matched against RDP database (Version 9) (Wang et al. 2007), and the fungal representative sequences were classified using the UNITE database (Version 7) (Koljalg et al. 2013). The alpha diversity statistics including Chao1, ACE and Shannon indexes were calculated for each sample with Mothur (Version v.1.30). Principal coordinates analysis (PCoA) and unweighted pair-group method with arithmetic mean (UPGMA) clustering were performed using R language, and the Bray–Curtis index was used as a distance measure.

### Statistical analysis

Multiple comparison was carried out by one-way analysis of variance (ANOVA) followed by LSD’s test (*P *< 0.05) using SPSS 19.0 software.

## Results

### Overall results of high-throughput sequencing

High-throughput sequencing of soil samples resulted in a total of 375,463 high-quality sequences for bacteria from the six soil samples with the effectiveness above 82% for all samples. Number of high-quality sequences per sample varied from 56,461 to 69,089 (mean = 62,577) (Additional file 1: Table S1). For fungi, 575,855 high-quality sequences were obtained from the six soil samples with the effectiveness above 90% for all samples. The number of high-quality sequences per sample varied from 65,607 to 114,088 (mean = 95,976) (Additional file 1: Table S2). The rarefaction curve of OTU for each library had approached a saturation plateau, indicating that the sequencing library had reached saturation and the results can truly reflect the sample condition (Additional file 1: Fig. S1).

**Fig. 1:**
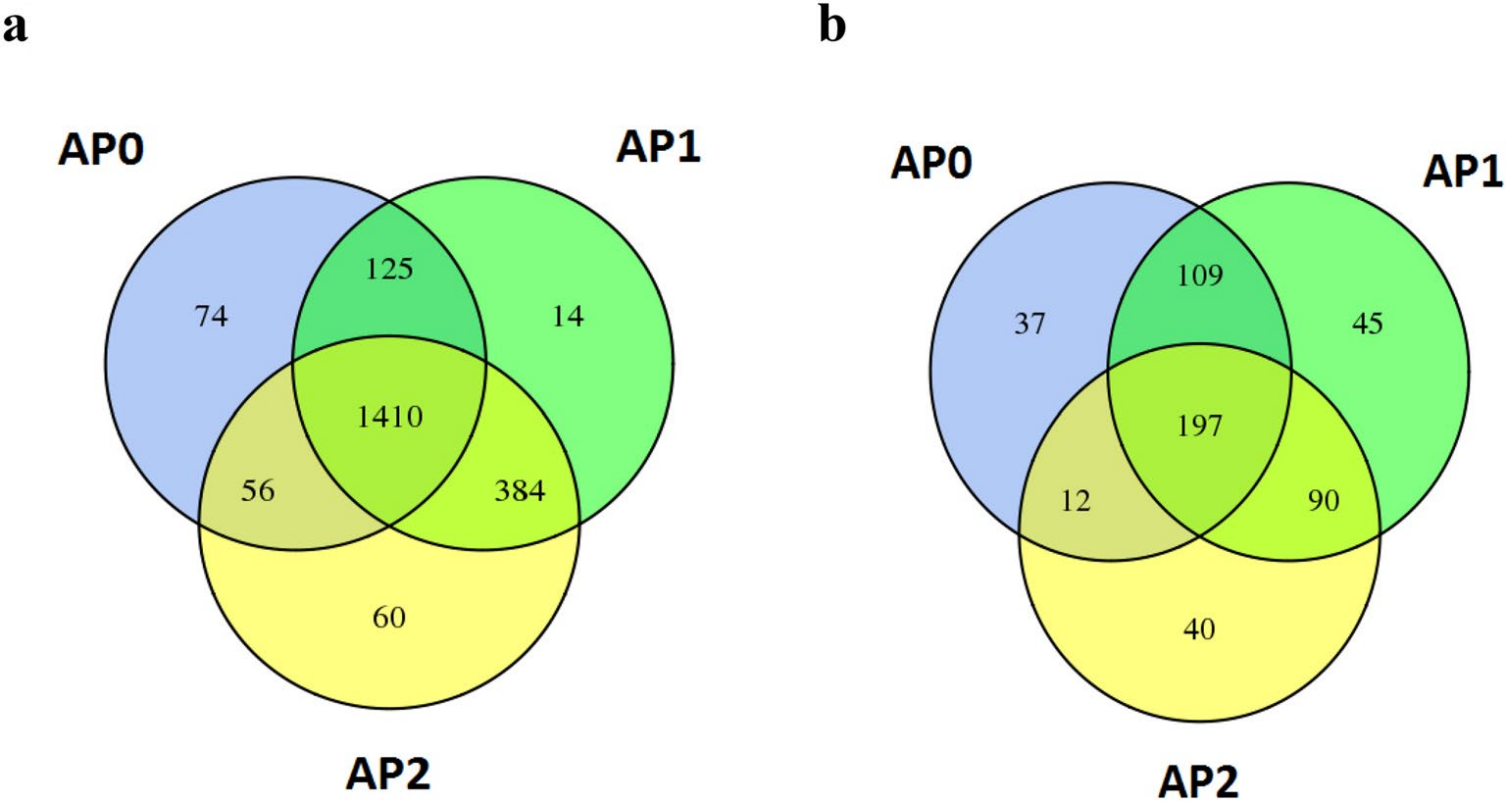
Copy numbers of bacteria (**a**) and fungi (**b**). AP0, AP1 and AP2 represent soil with continuously cropping histories of *Andrographis paniculata* for 0, 1 and 2 years

For bacteria, sequences across all samples were clustered into 2123 OTUs. OTU numbers increased about 15% once experienced replanting (AP0: 1665; AP1: 1933; AP2: 1910). Apart from 1410 OTUs (13.77%) shared by three groups of samples, 74, 14, and 60 unique OTUs were only found in AP0, AP1 and AP2, respectively (Fig. 1a). Fungi sequences across all samples were clustered into 530 OTUs, and OTU numbers ranked as AP1 (441) > AP0 (355) > AP2 (339). Among them, 197 OTUs were commonly identified in the three groups of samples; 37, 45, and 40 were only found in AP0, AP1 and AP2, respectively (Fig. 1b).

### Microbial alpha- and beta-diversities in soil continuously cropped *A. paniculata*

As showed in Table 1, both richness and diversity of bacteria increased after continuous cropping. As for fungi, ACE and Chao1 indexes were the highest in AP1. The Shannon index decreased with the increasing duration of the continuous cropping, indicating the fungal diversity in soil was reduced.

**Table 1:** Microbial richness and diversity of rhizospheric soil continuously cropped *Andrographis paniculata*

Group	Bacteria			Fungi		
	Richness estimators		Diversity indices	Richness estimators		Diversity indices
	ACE	Chao1	Shannon	ACE	Chao1	Shannon
AP0	1682.58	1711.13	5.44	328.50	332.25	4.13
AP1	1908.42	1849.90	5.90	403.02	402.77	3.92
AP2	1900.62	1929.57	6.40	306.02	307.25	3.25

**Fig. 2:**
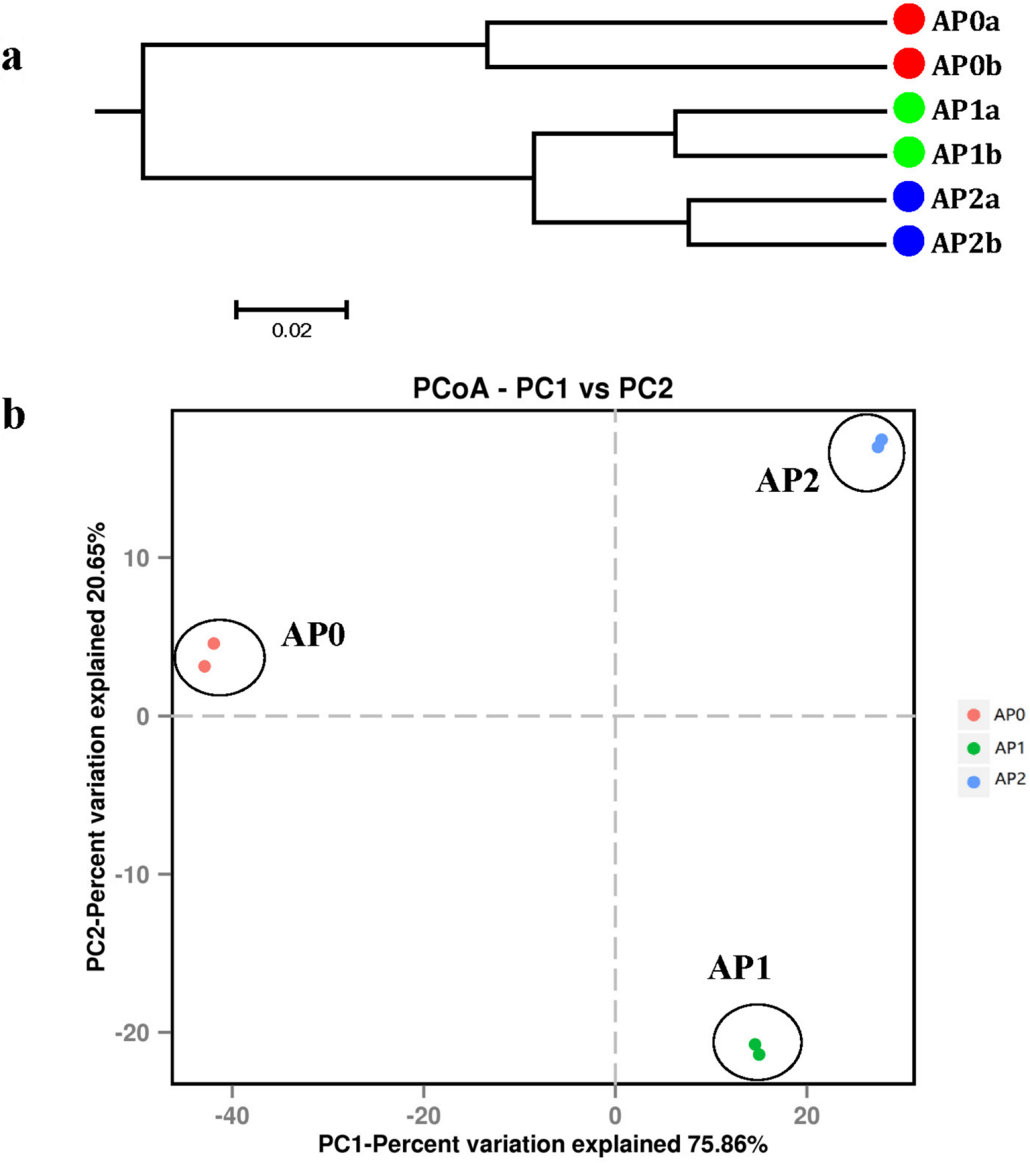
Unweighted pair-group method with arithmetic mean (UPGMA) dendrogram (**a**) and principal coordinate analysis (PCoA) (**b**) of bacterial communities based on Bray–Curtis distance. AP0, AP1 and AP2 represent soils with continuously cropping histories of *Andrographis paniculata* for 0, 1 and 2 years

**Fig. 3:**
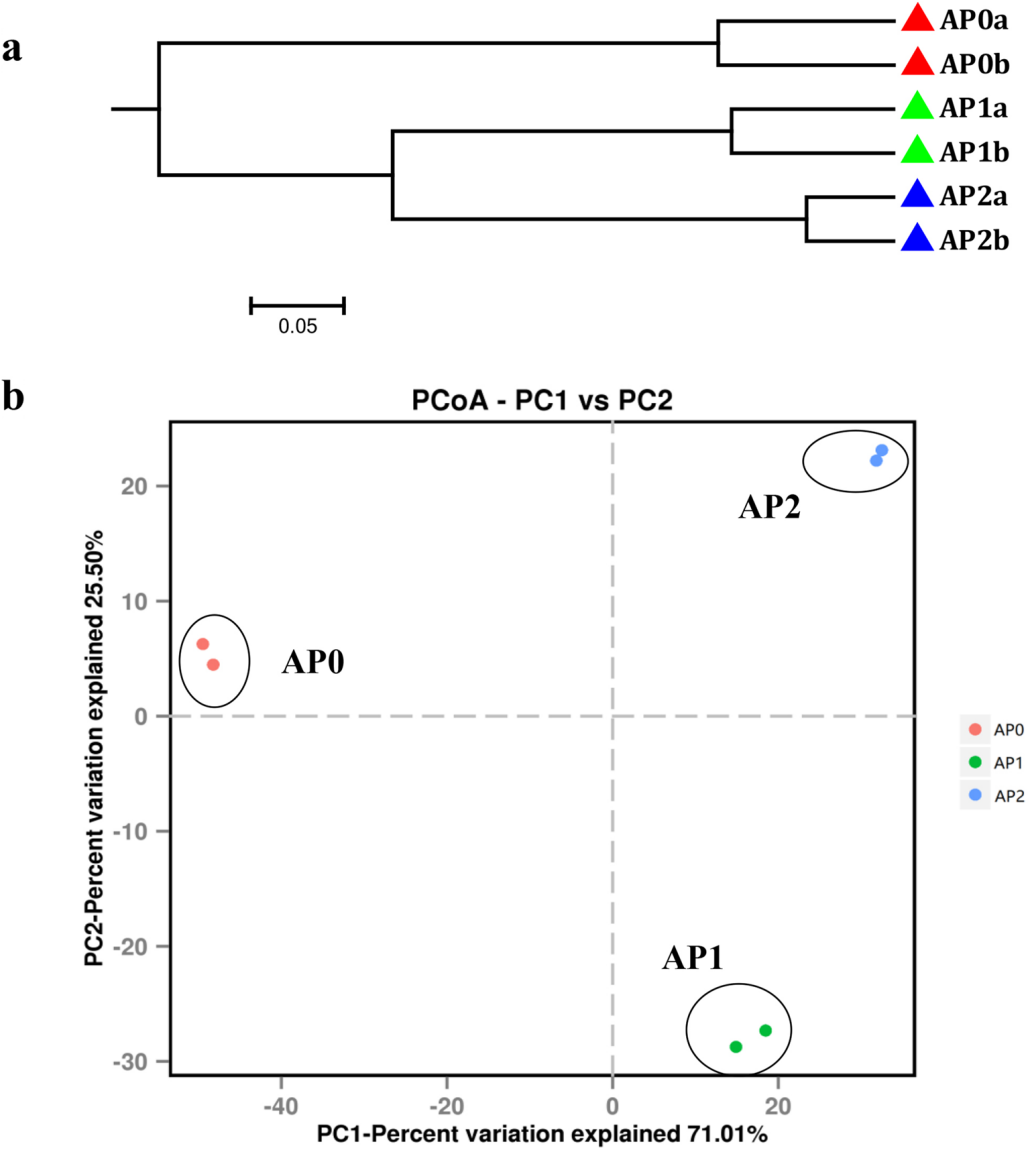
Unweighted pair-group method with arithmetic mean (UPGMA) dendrogram (**a**) and principal coordinate analysis (PCoA) (**b**) of fungal communities based on Bray–Curtis distance. AP0, AP1 and AP2 represent soils with continuously cropping histories of *Andrographis paniculata* for 0, 1 and 2 years

Furthermore, UPGMA clustering and PCoA were performed to investigate beta diversity patterns of microbial communities. UPGMA clustering results of bacterial (Fig. 2a) and fungal (Fig. 3a) communities both indicated that AP1 and AP2 soils clustered together, but were separated from AP0 soils. Concomitantly, PCoA also revealed distinct differences in community structures of bacteria (Fig. 2b) and fungi (Fig. 3b) between soil samples continuously cropped for different years. For bacterial community, the two main coordinates extracted (PC1 and PC2) explained 96.51% of the variation, of which PC1 explained 75.86% of the variations. As for fungal community, PC1 and PC2 explained 96.51% of the variations, of which PC1 explained 71.01% of the variations. AP1 and AP2 were grouped together and were clearly separated from AP0, indicating that AP1 and AP2 had similar microbial community structures on PC1. In addition, AP1 and AP2 were separated from each other along the PC2.

### Shifts of bacterial community composition in soil by continuous cropping

**Fig. 4:**
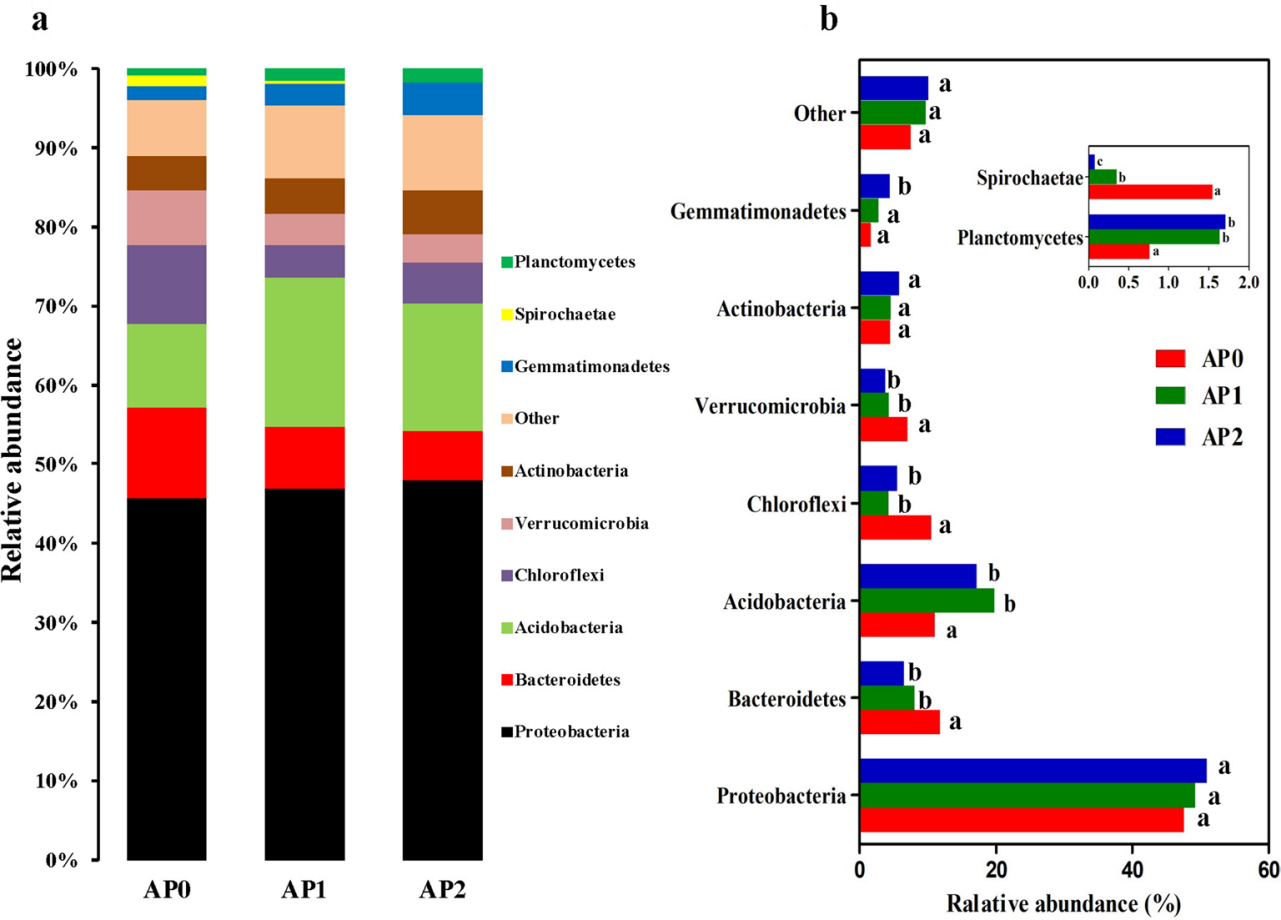
Relative abundance of the dominant bacterial phyla in the soils continuously cropped *Andrographis paniculata.***a** Bacterial community composition at the phylum level. **b** Variations of bacterial community composition. Only phyla with more than 1% relative abundance in at least one sample were shown and different letters mark significant difference at *P *< 0.05. The “Other” group contained the phyla with relative abundance < 1%. AP0, AP1 and AP2 represent soils with continuously cropping histories of *A. paniculata* for 0, 1 and 2 years

At the phylum level, the OTUs in all soil samples were primarily assigned to the 9 bacterial phyla (Fig. 4a). The dominant phyla (relative abundance > 10%) across all samples were *Proteobacteria*, *Acidobacteria* and *Bacteroidetes*, accounting for 70.4–77.1% of the bacterial sequences. In addition, *Chloroflexi*, *Verrucomicrobia*, *Actinobacteria*, *Gemmatimonadetes*, *Spirochaetae* and *Planctomycetes* were also present in all samples with a relative abundance between 1% and 10%. Members of *Proteobacteria* were detected in all soil samples at a minimum frequency of 47.63%, which increased with continuous cropping years, and reached the highest levels in AP2 (51.01%). The relative abundance of *Acidobacteria* and *Planctomycetes* increased considerably after continuous cropping (*P *< 0.05), while *Spirochaetae*, *Bacteroidetes*, and *Verrucomicrobia* significantly decreased (*P *< 0.05). Compared with AP0, *Gemmatimonadetes* abundance significantly increased in AP2 (*P *< 0.05). *Actinobacteria* abundance had no significant change in all samples (Fig. 4b).

**Fig. 5:**
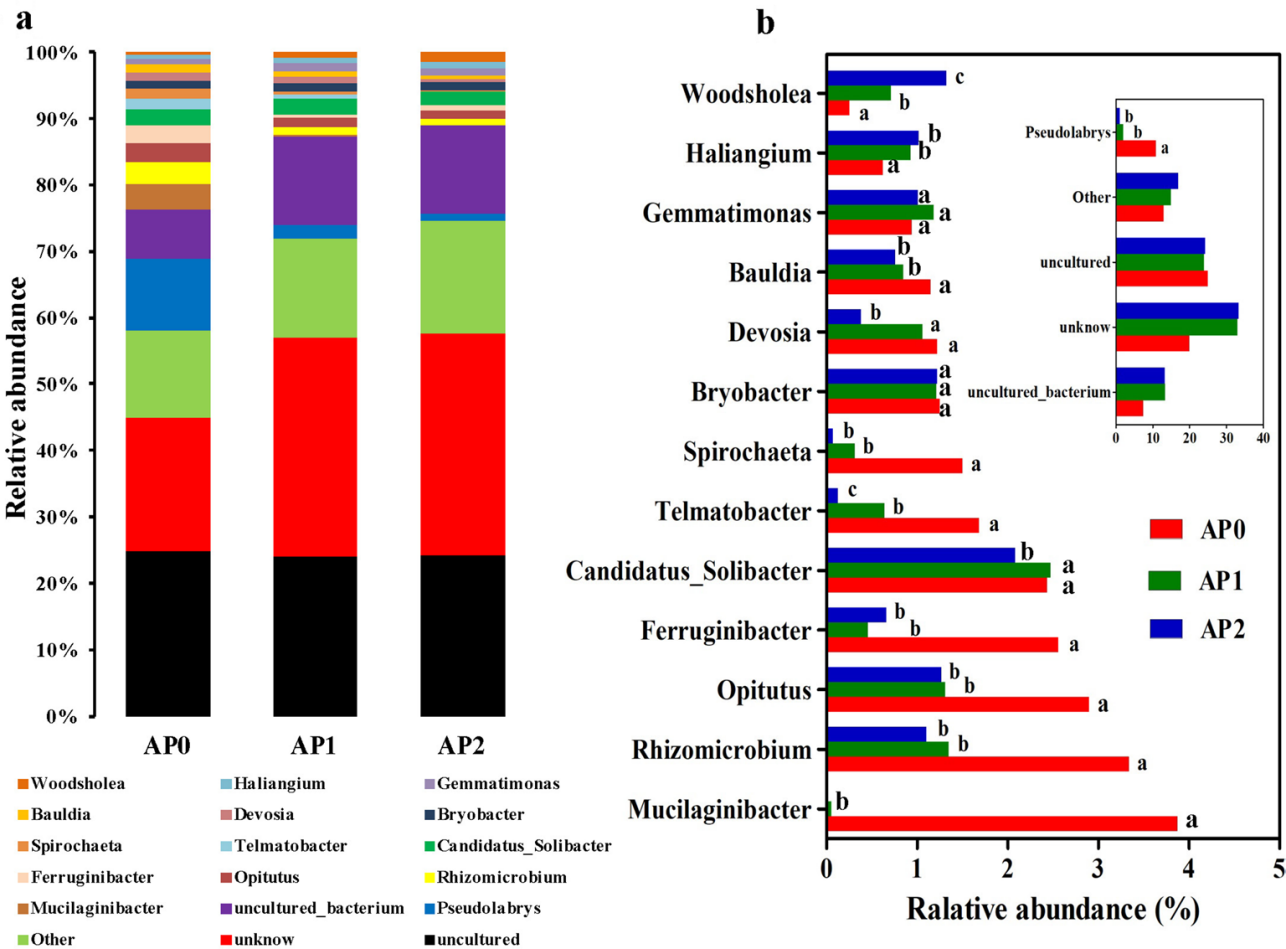
Relative abundance of the dominant bacterial genus in the soils continuously cropped *Andrographis paniculata.***a** Bacterial community composition at the genus level. **b** Variations of bacterial community composition. Only genera with more than 1% relative abundance in at least one sample were shown and different letters mark significant difference at *P *< 0.05. The “Other” group contained the genera with relative abundance < 1%. AP0, AP1 and AP2 represent soils with continuously cropping histories of *A. paniculata* for 0, 1 and 2 years

At the genus level, the classified OTUs were affiliated in 14 known genera with a relative abundance larger than 1% in at least one sample (Fig. 5a). OTUs including *Uncultured*, *Unknown* and *Uncultured bacterium* accounted for 52–70% of the bacterial sequences. The relative abundances of *Pseudolabrys*, *Bauldia*, *Spirochaeta*, *Telmatobacter*, *Ferruginibacter*, *Opitutus*, *Rhizomicrobium*, and *Mucilaginibacter* were remarkably decreased after continuous cropping (*P* < 0.05), while *Woodsholea* and *Haliangium* significantly increased (*P* < 0.05. Fig. 5b). Compared with AP0, the relative abundances of *Devosia* and *Candidatus*-*Solibacter* were significantly decreased in AP2 (*P* < 0.05).

### Shifts of fungal community composition in soil by continuous cropping

**Fig. 6:**
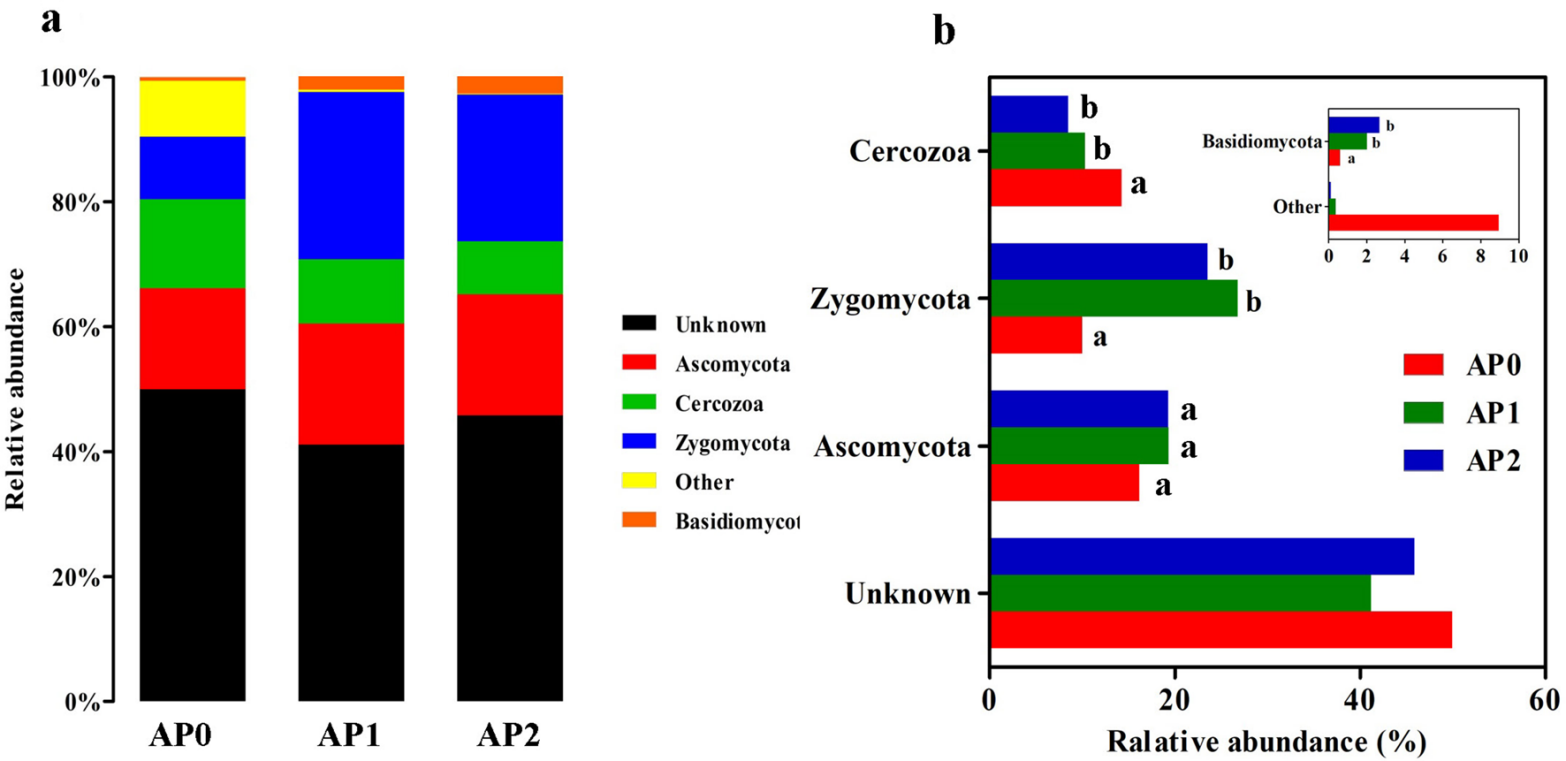
Relative abundance of the dominant fungal phyla in the soils continuously cropped *Andrographis paniculata.***a** Fungal community composition at the phylum level. **b** Variations of fungal community composition. Only phyla with more than 1% relative abundance in at least one sample were shown and different letters mark significant difference at *P *< 0.05. The “Other” group contained the phyla with relative abundance < 1%. AP0, AP1 and AP2 represent soils with continuously cropping histories of *A. paniculata* for 0, 1 and 2 years

At the phylum level, the OTUs classified from all soil samples were mainly affiliated in four fungal phyla with a relative abundance above 1% in at least one sample, including *Ascomycota*, *Cercozoa*, *Zygomycota* and *Basidiomycota* (Fig. 6a). Among them, *Ascomycota*, *Cercozoa*, and *Zygomycota* were the major phyla (relative abundance > 8%) across all the samples, accounting for 40.5–56.4% of the fungal sequences. *Basidiomycota* were also present in all samples with a relative abundance between 0.6% and 2.7%. In addition, over 40% of fungal sequences remained unknown across all the samples. As shown in Fig. 6b, after continuous cropping, the relative abundance of *Zygomycota* and *Basidiomycota* were significantly increased (*P* < 0.05) while *Cercozoa* significantly decreased (*P* < 0.05). There was no significant change of *Ascomycota* in all samples.

**Fig. 7:**
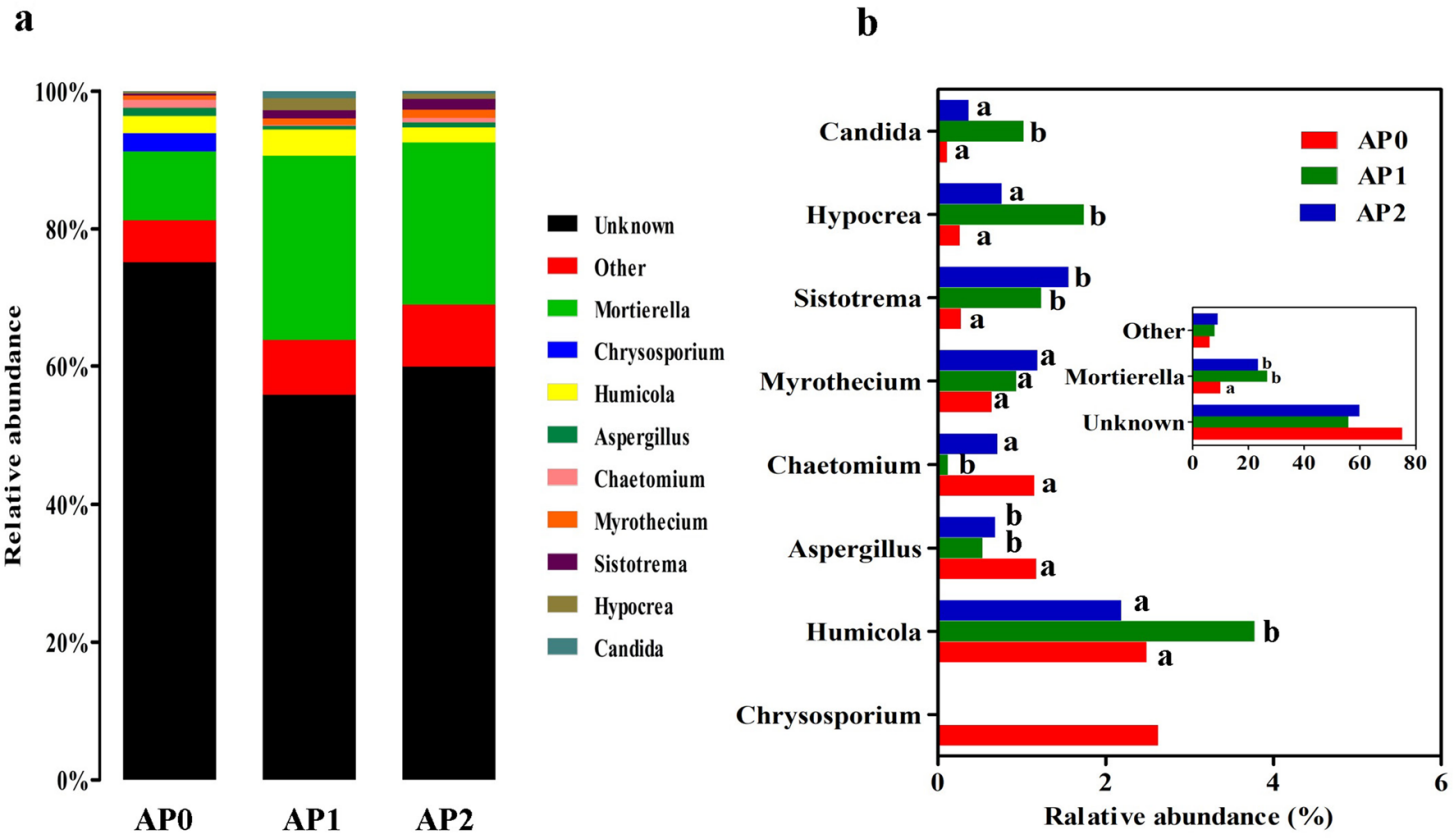
Relative abundance of the dominant fungal genus in the soils continuously cropped *Andrographis paniculata.***a** Fungal community composition at genus level. **b** Variations of fungal community composition. Only genera with more than 1% relative abundance in at least one sample were shown and different letters mark significant difference at *P *< 0.05. The “Other” group contained the genera with relative abundance < 1%. AP0, AP1 and AP2 represent soils with continuously cropping histories of *A. paniculata* for 0, 1 and 2 years

At the genus level, the classified OTUs were affiliated in nine known genera with a relative abundance larger than 1% in at least one sample, including *Mortierella*, *Chrysosporium*, *Humicola*, *Aspergillus*, *Chaetomium*, *Myrothecium*, *Sistotrema*, *Hypocrea* and *Candida* (Fig. 7a). Among them, *Mortierella* was the most abundant genus with a relative abundance over 10% in all samples, followed by *Humicola* with a relative abundance above 2% (Fig. 7b). There were 55.8–75.1% of fungal sequences remained unknown across all the samples. The relative abundances of *Mortierella* and *Sistotrema* were significantly increased after continuous cropping (*P* < 0.05), while *Aspergillus* significantly decreased (*P* < 0.05). Additionally, the relative abundance of *Candida*, *Hypocrea*, and *Humicola* were initially remarkably increased in AP1 (*P* < 0.05), and subsequently dropped in AP2 (*P* < 0.05). Moreover, *Chrysosporium* had a relative abundance above 2% in AP0, while it was not detected in the continuous cropping groups.

## Discussion

In the current study, the shifts of rhizospheric bacterial and fungal communities in *A. paniculata* continuous cropping soil were analyzed using Illumina MiSeq. Distinct differences in both bacterial and fungal community structures among the soil samples that newly planted (AP0) and continuous cropped *A. paniculata* plants (AP1 and AP2) were highlighted by PCoA and UPGMA. Cluster analysis of bacteria and fungi also supported the finding that the structure of the soil microbiome of *A. paniculata* was influenced by continuous cropping practices. High microbial diversity is generally considered as an important parameter for soil health (Chaparro et al. 2012). In this study, rhizospheric soil with 0, 1, and 2 years replanting history of *A. paniculata* were found varied in the richness and diversity of microorganism. For soil bacteria, the richness and diversity indices exhibited increasing trends after continuous cropping, which was aligned with previous studies on *Vanilla planifolia* and *R. glutinosa* while was contrary to American ginseng and *P. notoginseng* under continuous cropping (Dong et al. 2016; Xiong et al. 2017; Dong et al. 2017; Wu et al. 2018b). As for fungi, the richness indices firstly increased in 1-year soil while dropped in 2-year soil; and a similar trend was also found in rhizospheric soil fungal communities in tea orchards, in which the richness indices increased in 1-year soil and then kept decreasing in 10-and 20-year soils (Li et al. 2020). In addition, the fungal diversity indices decreased with the increasing of the continuous cropping time in this study, which accorded with previous study of *Atractylodes macrocephala* under continuous cropping while was contrary to that of *Pseudostellaria heterophylla*, American ginseng, and *P. notoginseng* (Wu et al. 2016; Dong et al. 2017; Tan et al. 2017; Zhu et al. 2020). Moreover, fungal diversity significantly decreased in the 10- and 20-year tea rhizospheric soils compared with that in the 1-year soil (Li et al. 2020). The discrepancy on microbial diversity between these different studies might be due to not only the longevity of monoculture but also the different soil environment conditions, plant types, rhizocompartment, etc.

The taxonomic analysis also revealed the variations of rhizospheric bacterial and fungal communities in soils under continuous *A. paniculata* cropping. In detail, *Proteobacteria*, *Acidobacteria*, *Bacteroidetes*, *Chloroflexi*, *Verrucomicrobia* and *Actinobacteria* were the most common bacterial phyla in the bacterial community of all samples. *Proteobacteria* was shown to be dominating the bacterial community of different geographic regions and soil types (Janssen 2006), members of this phylum may play a key role in phylogenetic, ecological and pathogenic values and participate in energy metabolism, such as the oxidation of organic and inorganic compounds (Zhou et al. 2019). In this study, *Proteobacteria* was the most abundant phylum, accounted for 49.3% of the average relative abundance, and did not change by different monoculture time spans. This result was in line with several studies that *Proteobacteria* was the most dominant phylum in continuous cropping soil of sweet potato (Li et al. 2019a), banana (Shen et al. 2018), and vanilla (Xiong et al. 2015b). However, *Proteobacteria* abundance was shown significantly reduced in potato (Liu et al. 2014) and black pepper (Xiong et al. 2015a), while increased in tobacco (She et al. 2017) and wheat (Sanguin et al. 2009) after continuous cropping. Root exudates and plant species play key roles in shaping the rhizospheric bacterial composition, which result in different plant genotype-specific community structures even in the same soil (Grayston et al. 1998; Marschner et al. 2004; Chaparro et al. 2012). Therefore, these discrepancies in *Proteobacteria* abundance could be partly attributed to the different plant species. On the other hand, they might also be attributed partly to the wide range of bacteria in *Proteobacteria* phylum and the results could not comprehensively reflect the alteration in populations at lower taxonomic levels (Sanguin et al. 2009). *Acidobacteria* are also ubiquitous and abundant members of soil bacterial communities (Janssen 2006), and play important roles in organic matter decomposition and nutrient cycles (Eichorst et al. 2018). In the current study, the relative abundance of *Acidobacteria* increased considerably in soil after continuous cropping with *A. paniculata*, which accorded with soils that long-term continuously cropped black pepper and tea (Xiong et al. 2015a; Li et al. 2016b). Fierer et al. suggested that *Acidobacteria* were most abundant in soils with very low resource availability (Fierer et al. 2007); continuous cropping practices could commonly decrease the soil nutrition contents, this may explain in part why the higher relative abundances of *Acidobacteria* were detected in soil after continuous *A. paniculata* cropping. *Actinobacteria* was consistently associated with pathogen antagonism, and was found strongly enriched in suppressive soils (Mendes et al. 2011; Xiong et al. 2017) and decreased markedly along the years of consecutive cropping (Xiong et al. 2015b). In this study, *Actinobacteria* was present in all samples with no significant change in relative abundance. At the genus level, there were 52–70% of the bacterial sequences corresponded to uncultured and unknown bacteria, a high percentage of unclassified sequences also have been reported in the rhizosphere of other plants such as maize (Correa-Galeote et al. 2016). Apart from the uncultured and unknown bacteria, *Pseudolabrys*, *Rhizomicrobium*, *Candidatus_Solibacter*, *Opitutus* and *Bryobacter* were the dominant genera across all samples. *Pseudolabrys* are ubiquitous in hydrocarbon-rich soil, and had been identified as hydrocarbon degraders (Miao et al. 2019). In this study, *Pseudolabrys* was the most abundant genus; the maximum abundance was up to 10.88% in AP0 while remarkably decreased after continuous cropping. Additionally, *Rhizomicrobium* and *Opitutus* were also reduced after continuous cropping, while there were no obvious change in *Candidatus_Solibacter* and *Bryobacter*. *Mucilaginibacter* was found in AP0 with a relative abundance of 3.88%; however, after continuous cropping, it could hardly be observed. Taken together, these findings profiled the variations of rhizospheric bacterial communities in soil continuous cropped *A. paniculata*.

Regarding the fungi in soil continuous cropped *A. paniculata*, taxonomic analysis revealed that fungal sequences were mainly classified into four known phyla including *Ascomycota*, *Zygomycota*, *Cercozoa* and *Basidiomycota*, and the first three were the predominant phyla with an average relative abundance above 10% in all samples. *Zygomycota* was the most abundant phylum and markedly increased after consecutive *A. paniculata* monoculture, which was consistent with findings in soybean under continuous cropping (Liu et al. 2019). *Zygomycota* is a saprophytic phylum and plays important roles in decomposing plant debris; the increasing abundance in continuous cropping soil might promote the decomposition of *A. paniculata* debris. Xiong et al. also found significant enrichment of phylum *Zygomycota* in suppressive soil associated with *Fusarium* wilt disease in vanilla long-term continuous cropping system, suggesting a disease suppressive role of the dominant *Zygomycota* species (Xiong et al. 2017). *Ascomycota* and *Basidiomycota* are commonly observed in soil. In this study, the relative abundance of *Ascomycota* showed an increasing trend after continuous cropping, which was consistent with that in rhizospheric soil of black pepper (Li et al. 2016c) and *R. glutinosa* (Wu et al. 2018a) under continuous cropping system while contrary to that in potato continuous cropping soil (Gao et al. 2019). In addition, *Basidiomycota* also increased after continuous cropping and reached the maximum abundance of 2.68% in AP2, which was contrary to the previous study in potato (Gao et al. 2019). Moreover, *Cercozoa* phylum had the maximum abundance of 14.3% in AP0, and showed a gradual downtrend in abundance after continuous cropping of *A. paniculata*. At genus level, *Mortierella*, *Humicola* and *Sistotrema* were the top three abundant genera with average relative abundance above 1% across all samples. Among them, *Mortierella*, belonging to phylum *Zygomycota*, was the most abundant fungal genus and accounted for 10%–26.8% of fungal sequences. Our results revealed that consecutive *A. paniculata* monoculture significantly increased the relative abundance of *Mortierella*, which was agreed with the continuous cropping studies of other plants such as *P. heterophylla* (Wu et al. 2016) and soybean (Liu et al. 2019). *Mortierella* appeared to participate in plant pathogens suppression, which effectively inhibited occurrence of Chinese cabbage clubroot disease (Narisawa et al. 1998), as well as protected banana (*Musa* sp.) from *Fusarium* wilt disease and *Fusarium* root rot (Shen et al. 2018). Therefore, the increasing abundance of *Mortierella* in this study might help *A. paniculata* adapt to the continuous cropping practice for its own growth, protect them from some diseases. *Sistotrema* and *Myrothecium* also showed increasing trends in relative abundance, while *Aspergillus* was decreased after continuous cropping. *Chrysosporium* that had a relative abundance > 2% in AP0, was undetectable in continuous cropped soil. All these results indicated that consecutive *A. paniculata* cropping influenced the structure and composition of soil fungal community.

Numerous studies have confirmed that cropping system changed the composition of soil microorganisms. For instance, microbial communities of rhizosphere soils significantly changed during the continuous cultivation of ginseng (Ying et al. 2012). Fungal populations exhibited significant dynamic changes in the continuous cropping of peanut, and both pathogenic and beneficial fungi were positively selected over time (Chen et al. 2012). Apparently, continuous *A. paniculata* cropping practices also changed the diversity and composition of rhizosphere soil microbial communities in this study. Interactions between plant roots and soil microorganisms are critical for plant fitness in natural environment (Zhalnina et al. 2018); plant species is thought to select specific microbial populations in the rhizosphere and root exudates are part of the driving force (Dong et al. 2017). Plant roots can exude a variety of compounds into the soil, including carbohydrates, amino acids, organic acids and secondary metabolites by diffusion, which will shape the microbial communities (Sasse et al. 2018). The compositions of root exudates are diverse and dynamic, depending on plant species, development stage, root traits, and environmental conditions (Chaparro et al. 2014; Mönchgesang et al. 2016; Sasse et al. 2018). *A. paniculata* is rich in terpenoids, flavonoids, and phenolics, which were identified as powerful allelochemicals and have been widely accepted as mediators that act between inter- and intraspecific root (Singh et al. 2003; Bertin et al. 2003; Bais et al. 2004; Weston et al. 2012). Aqueous extracts from soil continuously cropped *A. paniculata* could significantly inhibit its seed germination and seedlings’ root growth (Zeng et al. 2011; Li et al. 2016a; Hu et al. 2018). Zeng et al. found that *A. paniculata* released some alleochemicals into the soil during the growth period, and andrographolide was one of the main compounds (Zeng et al. 2011). Furthermore, Hu et al. confirmed this finding, as not only andrographolide but also two phenolic compounds (ferulic acid, caffeic acid) were abundant in 2-years old monoculture soil while were absent in uncultivated soil (Hu et al. 2018). Lowered andrographolide, dehydroandrographolide, and flavonoids contents were found in plants grown on soil continuously cropped with *A. paniculata* (Li et al. 2019b). Taken together, continuous cropping practices might facilitate the release process thus resulted in less accumulation in the plants (Li et al. 2017; 2019b). These released compounds in rhizosphere soil further affected the rhizospheric soil microbiome diversity and composition. It has also been shown that some of released compounds can attract beneficial microorganisms and affect the assembly of rhizosphere microbiomes that enhance the capacity of plants to adapt to their environment (Bulgarelli et al. 2013), which might partly explain the increasing yield of *A. paniculata* in our previous continuous cultivation study (Li et al. 2017). In conclusion, we hypothesized that continuous cropping practice of *A. paniculata* promoted release of compounds into the soil, which changed the rhizospheric microbial diversity and composition; these changes in the microbial community could disturb the soil micro-ecology and thus affect *A. paniculata* growth under continuous cropping system. However, further studies are required to illuminate the precise mechanism.

## Conclusions

High-throughput sequencing analyses provided simultaneous and detailed insights into the changes of community structure and diversity of bacteria and fungi in the continuous cropping system of *A. paniculata*. Our findings will help understanding of soil micro-ecological environments in the rhizosphere of *A. paniculata* under continuous cropping system, and provide insight into the better theoretical reference for the better cultivation and management of *A. paniculata.*

## Supplementary information

**Additional file 1: Table S1.** Sequencing output of soil bacterial sequences. **Table S2.** Sequencing output of soil fungal sequences. **Fig. S1.** Rarefaction curves of soil microbial communities based on OTUs at the 97% similarity cut-off level for individual samples. (a) and (b) are the rarefaction curves of OTU for bacterial and fungal communities, respectively. AP0, AP1 and AP2 represent soil with continuously cropping histories of *A. paniculata* for 0, 1 and 2 years. “a” and “b” mean the two replicates.

## Data Availability

Sequence data generated in this study have been deposited in the NCBI Sequence Read Archive (SRA) database (https://submit.ncbi.nlm.nih.gov/subs/sra/) under the BioProject of PRJNA605784 (Accession numbers SRR11058251-SRR11058253 and SRR11058254-SRR11058256).

## Abbreviations

ANOVA: One-way analysis of variance
OTU: Operational taxonomic unit
PCoA: Principal coordinates analysis
UPGMA: Unweighted pair-group method with arithmetic mean
