# Supplementary material for: Variations of rhizospheric soil microbial communities in response to continuous Andrographis paniculata cropping practices
Source: Bot Stud. 2020 Jun 15;61:18. doi: 10.1186/s40529-020-00295-1 (PMC7295922; doi:10.1186/s40529-020-00295-1)
Supplement: Supplementary file 1 — Additional file 1: Table S1. Sequencing output of soil bacterial sequences. Table S2. Sequencing output of soil fungal sequences. Fig. S1. Rarefaction curves of soil microbial communities based on OTUs at the 97% similarity cut-off level for individual samples. (a) and (b) are the rarefaction curves of OTU for bacterial and fungal communities, respectively. AP0, AP1 and AP2 represent soil with continuously cropping histories of A. paniculata for 0, 1 and 2 years. “a” and “b” mean the two replicates. [file 40529_2020_295_MOESM1_ESM.docx]

**Table S1.** Sequencing output of soil bacterial sequences

| Sample_ID | PE_Reads | Raw_Tags | Clean_Tags | AvgLen(bp) | GC(%) | Q20(%) | Q30(%) | Effective(%) |
| --- | --- | --- | --- | --- | --- | --- | --- | --- |
| AP0-1 | 66685 | 63779 | 56461 | 454 | 54.6 | 96.47 | 93.29 | 84.67 |
| AP0-2 | 79106 | 77333 | 68166 | 453 | 54.96 | 97.86 | 95.89 | 86.17 |
| AP1-1 | 71436 | 67914 | 59218 | 456 | 55.57 | 96.17 | 92.69 | 82.9 |
| AP1-2 | 66788 | 65154 | 57857 | 456 | 55.91 | 97.71 | 95.57 | 86.63 |
| AP2-1 | 77642 | 73823 | 64672 | 457 | 56.01 | 96.45 | 93.18 | 83.3 |
| AP2-2 | 79522 | 77684 | 69089 | 456 | 56.43 | 97.74 | 95.57 | 86.88 |

**Table S2.** Sequencing output of soil fungal sequences

| Sample_ID | PE_Reads | Raw_Tags | Clean_Tags | AvgLen(bp) | GC(%) | Q20(%) | Q30(%) | Effective(%) |
| --- | --- | --- | --- | --- | --- | --- | --- | --- |
| AP0-1 | 101723 | 93697 | 92107 | 345 | 41.75 | 97.82 | 96.09 | 90.55 |
| AP0-2 | 72639 | 66856 | 65607 | 345 | 42.75 | 98.6 | 97.67 | 90.32 |
| AP1-1 | 118767 | 114501 | 113433 | 306 | 44.26 | 98.31 | 96.92 | 95.51 |
| AP1-2 | 97751 | 94381 | 93624 | 313 | 44.17 | 98.82 | 98 | 95.78 |
| AP2-1 | 118437 | 114789 | 114088 | 292 | 44.56 | 98.86 | 97.95 | 96.33 |
| AP2-2 | 99496 | 97363 | 96996 | 302 | 49.73 | 99.11 | 98.7 | 97.49 |


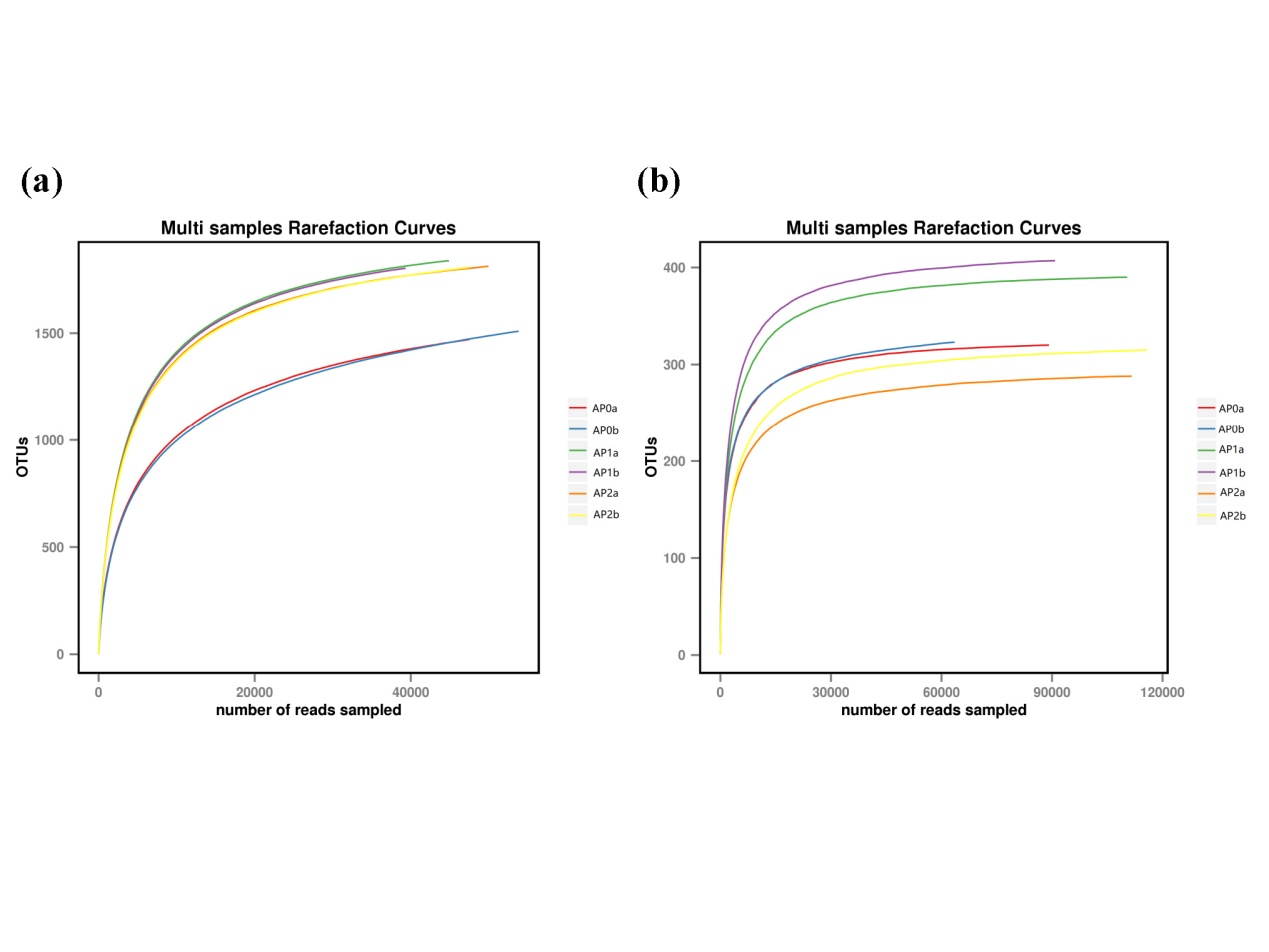


**Fig. S1.** Rarefaction curves of soil microbial communities based on OTUs at the 97% similarity cut-off level for individual samples. (a) and (b) are the rarefaction curves of OTU for bacterial and fungal communities, respectively. AP0, AP1 and AP2 represent soil with continuously cropping histories of *A. paniculata* for 0, 1 and 2 years. “a” and “b” mean the two replicates.
